# Elaboration and Characterization of Bioactive Films Obtained from the Incorporation of Cashew Nut Shell Liquid into a Matrix of Sodium Alginate

**DOI:** 10.3390/antiox10091378

**Published:** 2021-08-28

**Authors:** Larruama Vasconcelos, Marthyna de Souza, Juliana de Oliveira, Edson Silva Filho, André Silva, Selma Elaine Mazzetto, Elzânia Sales Pereira, Ronaldo Lopes Oliveira, Leilson Bezerra

**Affiliations:** 1 Department of Animal Science, Animal Health and Science Graduate, Federal University of Campina Grande, Avenida Universitária, s/n-Jatobá, Patos 58708110, Brazil; juli.foo@gmail.com (J.d.O.); andre.leandro@ufcg.edu.br (A.S.); 2 Department of Animal Science, Federal University of Piaui, Ininga, s/n, Teresina 64049550, Brazil; edsonfilho@ufpi.edu.br; 3 Department of Organic and Inorganic Chemistry, Federal University of Ceará, Contorno Street, Fortaleza 60451970, Brazil; selma@ufc.br; 4 Department of Animal Science, Federal University of Ceará, Av. Mister Hull, Fortaleza 60356000, Brazil; elzania@ufc.br; 5 Department of Animal Science, Federal University of Bahia, Av. Adhemar de Barros, 500, Ondina, Salvador 40170110, Brazil; ronaldooliveira@ufba.br

**Keywords:** *Anacardium occidentale*, edible film, bioactive packaging, antioxidant film, sustainability

## Abstract

The objective of this work was to obtain and characterize sodium alginate-based biopolymer films with the addition of cashew nut shell liquid (CNSL). The study employed a completely randomized design, including 0%, 0.5%, 1%, and 1.5% inclusion of CNSL. Uniform formation of the films was observed, and the addition of CNSL provided better thermal resistance than did the treatment without inclusion, while the addition of CNSL reduced the homogeneity of the microstructure, especially for the 1.5% inclusion level. The permeability of the film increased as the level of CNSL increased, especially in response to the concentrations of 1% and 1.5%, and no significant difference in permeability was observed between these treatments. The tensile strength decreased proportionally as a function of the addition of CNSL, as its inclusion increased the elasticity and elongation of the films. In addition, the films with CNSL demonstrated strong antioxidant activity and discrete antimicrobial activity, and ecotoxicity analysis showed that the levels of CNSL tested and the films produced were nontoxic. Thus, these films are promising and self-sustainable alternatives for the agrifood industry.

## 1. Introduction

Food packaging plays a fundamental role both in food preservation and throughout its distribution chain [1]. The main objective is to separate food from the circulating environment, decreasing contact with various conditions leading to deterioration, including microorganisms, oxygen, pH, water vapor, and odious flavors, as well as preventing losses of important compounds such as volatiles; in short, packaging protects the food from contaminants and consequently prolongs the useful life of the food [2]. In this context, the material used in the manufacturing and manipulation of packaging is very important in preserving the quality of food and delaying its decomposition rate [3].

Much of the food packaging currently used was developed using petroleum-based polymers and plastics, in view of the lower financial cost, high mechanical strength and rigidity of these materials, as well as their versatility of shapes and heat sealing capacity. As a result, these materials occupy 37% of the total market for food packaging materials. However, this type of packaging is nonrenewable and nonbiodegradable and is responsible for an increase in environmental pollution; as such petroleum-based polymers and plastics as environmentally are considered unfavorable [4,5].

Thus, the increase in research involving eco-friendly packaging, such as the production of biodegradable films, has recently gained greater traction [6]. This is because these films can replace plastic bags used on food products because they have a lifetime of few minutes to hours, while the plastics stay in the environment for decades [7].

Films obtained from biopolymeric matrices have been gaining prominence since they are biodegradable and can often delay the deterioration of food [8]. Different biopolymers can be used for the fabrication of these films, including polysaccharides, which are common materials recently used to obtain films with biodegradability properties [9]. Among these biodegradable films, alginate has been widely used since films based on this biopolymer have excellent barrier properties against oxygen, carbon dioxide, and lipids, in addition to excellent mechanical characteristics, including tensile strength and ductility [10].

Nevertheless, unfortunately, there is no natural polymer that, individually, is capable of providing all the desired properties for film formation, namely, moisture barrier properties, mechanical and thermal properties, ability to form coatings and gelling properties, water-solubility, odorlessness, tastelessness, colorlessness, translucence, flexibility, and possessing antimicrobial activity and antioxidant activity while preserving food additives such as antioxidants, dyes, and flavors, among others. Thus, it is necessary to select and integrate the ingredients of films so that they act synergistically to obtain films with all the properties consistent with their intended use [10].

Therefore, one of the recent focuses in the fabrication of films is to increase their functionality, adding characteristics such as antioxidant and/or antimicrobial activity, by incorporating components with these properties, for instance, functional oils that can increase the stability of food during storage, preventing food deterioration by delaying lipid oxidation, as well as the growth of pathogenic microorganisms [11], especially in easily perishable foods.

Researchers consider the importance of antioxidants derived from low-cost renewable sources, for example, cashew nut shell liquid (CNSL), which is a cheap oil derived from byproducts of the cashew (*Anacardium occidentale* L.) and composed mainly of anacardic acid (60% to 65%) [12]. For the removal of the nut, the cashew is submitted to an industrial process through submission to high temperatures, in which anacardic acid undergoes a decarboxylation reaction, generating technical CNSL, composed mainly of cardanol (60% to 65%), cardol (15% to 20%), polymeric constituents (10%) and 2-methyl cardol (traces). This product of the high-temperature treatment presents as a dark (almost black), viscous liquid with a characteristic odor [13]. CNSL, in addition to being a renewable, biodegradable, inexpensive source in abundance in Brazil, is extremely rich in phenolic and bioactive compounds, thus presenting powerful antioxidant activity, especially due to the high content of cardanol present in technical CNSL, in addition to antimicrobial activity [14]. Thus, CNSL exhibits desirable essential characteristics for use as an additive in the production of films aimed at slowing the lipid oxidation process and consequently increasing the shelf life of food because the incorporated active agents define the functionality of packaging materials [15].

In this context, considering the environmental appeal of CNSL as the main focus of this study, as well as the importance of packaging, the sustainability of CNSL, and its potential to increase the shelf life of perishable foods, the present research aimed to develop and characterize bioactive films based on sodium alginate enriched with CNSL in order to identify their potential use in the agrifood industry as a possible substitute for nondegradable food packaging.

## 2. Materials and Methods

### 2.1. Material

Sodium alginate was purchased from Dinâmica Química Contemporânea LTDA (Indaiatuba, São Paulo, Brazil). The technical CNSL (containing 84.4% cardanol and 15.6% cardol) used was supplied by Amêndoas do Brasil LTDA (Fortaleza, Brazil). Brain heart infusion (BHI) agar and solvents were purchased from Merck (Darmstadt, Germany). Diammonium 2,2′-Azino-bis(3-ethylbenzothiazoline-6-sulfonate) (ABTS) and 2,2-Diphenyl-1-picryhydrazyl (DPPH) were purchased from Sigma-Aldrich (St. Louis, MO, USA). All chemicals were analytical grade.

### 2.2. Elaboration of the Biopolymer Films

Sodium alginate-based films were developed based on Oussalah et al. [16]. Initially, a sodium alginate solution was obtained at a concentration of 3% (*w*/*w*). Subsequently, glycerol and Tween 80 were added to the sodium alginate solution at concentrations of 2 and 0.5% (*w*/*w*, based on the alginate solution mass). The mixture was then heated to 70 °C on a hot plate and shaken with a glass rod for 1 h for total homogenization of the filmogenic solution (FS). Then, technical CNSL was added to the filmogenic solution at the respective inclusion levels (0%, 0.5%, 1.0%, and 1.5% (*w*/*w*, based on FS mass)), remaining under manual stirring (glass rod) for 20 min until total homogenization.

Finally, the mixture was placed into disposable Petri dishes (90 × 15 mm), properly identified, and dried in an air circulation oven at 45 °C for 24 h. After drying, the films were removed using tweezers, wrapped in aluminum foil, and stored in polyethylene Ziploc bags at 25 °C and 54% relative humidity [obtained using a saturated solution of Mg(NO_3_)_2_·6H_2_O].

### 2.3. Characterization of Films

#### 2.3.1. Thermogravimetric Analysis (TGA) and Differential Scanning Calorimetry (DSC)

The thermogravimetric and DSC curves were obtained via a simultaneous thermal analyzer (SDT Q600 V20.9 Build 20), with a N_2_ atmosphere (flow of 100 mL/min and heating of 10 °C/min), from 30 to 600 °C (30 to 300 °C was considered for DSC curves), using a platinum crucible containing 8 mg ± 0.0001 g of sample.

The parameters extracted from the thermogravimetric and DSC curves were the initial thermal degradation temperature—T_onset_ (extrapolated temperature at the point of intersection of the starting-mass baseline and the tangent to the thermogravimetric curve at the point of maximum declivity) and thermal degradation peak temperature, respectively, using OriginPro 8.

#### 2.3.2. Color and Opacity

Five measurements were randomly performed on each 90 × 15 mm size film replica, and the test was conducted in triplicate, resulting in fifteen readings for each film studied. The average of the measurements was considered. A digital colorimeter (CR 400; Minolta, Japan) calibrated with the white standard illuminant C was used to measure color and opacity. The parameters determined were luminosity or L* (L* = 0 [black] and L* = 100 [white]), red or a* (−a* = green and + a* = red), and yellow or b* (−b* = blue and + b* = yellow). The saturation index or chroma (C*) was calculated according to the following formula: (a*2 + b*2)0.5. [17]. The opacity (Y) was determined using the following equation: Y (%) = (Yp/Yb) × 100, where (Yp) is the opacity of the coating in the black pattern and (Yb) is the relative opacity in the white pattern [8].

#### 2.3.3. Morphology of the Films

The morphology was analyzed by scanning electron microscopy (SEM) using a TESCAN VEGA3 microscope (Brno, Czech Republic), corresponding to a tungsten thermionic emission system suitable for high and low vacuum operation, with an accelerating voltage from 5 kV. Before obtaining the micrographs, samples of all films (10 × 10 mm) were first metalized with a thin layer of gold.

#### 2.3.4. Thickness

A digital micrometer (Digimess, São Paulo, Brazil) was used to study the thickness of the films, measured at five different points of each film, chosen at random, in triplicate, thus totaling fifteen readings for each type of film, after the measurement of color and opacity, using the same 90 × 15 mm samples.

#### 2.3.5. Water Vapor Permeability

Water vapor permeability (WVP) was determined by gravimetric analysis according to Souza et al. [8]. Initially, the top of a permeation cell with distilled water (relative humidity of 100%, steam pressure of 2337 Pa and 20 °C) was sealed with the film and then placed in a desiccator with silica at 25 °C, 0% relative humidity, and a water vapor pressure of 0 Pa. Eventually, the cells were weighed every 2 h for 10 h. Linear regression was used to obtain the inclination of the loss of mass versus time. WVP was calculated as follows: WVP = (WVTR × T)/ΔP, where WVTR is the rate of water vapor transmission (g/m^2^·s), T is the average thickness (m), and ΔP is the partial difference of water vapor pressures (Pa) on both sides of the film.

#### 2.3.6. Mechanical Properties

The tests for maximum tensile strength, modulus of elasticity, and elongation at break were performed using a Universal Testing Machine (EMIC-DL-500) according to ASTM D882-12 standards (Standard Test Method for Tensile Properties of Thin Plastic Sheeting) [18]. The initial separation of the claws was 10 mm, and the traction speed was 5 mm/min. Software was used for data collection regarding the stress-strain curve (MPa) versus deformation (%). Young’s modulus for the samples was determined from the tangent of the elastic region of the stress-strain × deformation curves. Elongation values at breakage were estimated considering the ratio of final length at the point of the sample breakage to the initial length of a sample (10 mm) and expressed in %.

Ten repetitions were conducted for each sample with a length of 50 mm and a width of 10 mm. Before the analyses, the samples were maintained for 2 days at 25 °C and 50% relative humidity.

#### 2.3.7. Antimicrobial Activity of the CNSL

Strains of *Salmonella typhimurium* (ATCC 10028), *Listeria monocytogenes* (ATCC 7644), *Pseudomonas aeruginosa* (ATCC 8626), *Staphylococcus aureus* (ATCC 6538), *Bacillus cereus* (ATCC 14579), and *Escherichia coli* (ATCC 8739) were cultivated on brain heart infusion (BHI) agar. After incubation, the colonies of each lineage were aseptically placed into a sterile saline solution (NaCl 0.85%); a value of 0.5 on the McFarland scale (106 UFC/mL) was used to standardize the turbidity of the solutions.

The minimum inhibitory concentration (MIC) and the minimum bactericidal concentration (MBC) of CNSL were determined according to the Clinical and Laboratory Standards Institute [19] by the microdilution method in a 96-well microplate with concentrations ranging from 2 to 1024 μg/mL by serial dilutions. Then, 100 μL of BHI 10 broth (106 UFC/mL of each strain) was added to each well. A rotary shaker (150 rpm) was used to incubate the microorganisms at 37 °C for 24 h, and then aliquots of the wells without turbidity were transferred to Petri dishes with agar BHI medium and incubated at 37 °C for 24 h. The MBC was classified as the lowest concentration in which any microbial growth was not observed after the culture. The MIC was considered the highest concentration where turbidity in liquid medium was not observed, but growth was observed after cultivation in solid medium. Azithromycin and sterile saline solution were used as positive and negative controls, respectively.

#### 2.3.8. Antibacterial Activity of the Films—Agar Well Diffusion Method

The antibacterial activity of alginate films with and without CNSL was evaluated using the agar well diffusion method [8].

The sowing of the standard inoculum (0.5 on the McFarland scale) was carried out in the form of a carpet on the surface of Petri dishes with half BHI agar using a Drigalski spatula. Then, 5.0 mm diameter wells were made on each plate and were filled with 20 μL of each filmogenic solution with different concentrations of CNSL. As positive and negative controls, azithromycin (1028 µg/mL) and autoclaved distilled water were used, respectively. The incubation conditions of the inoculated plates were 37 °C for 24 h. The diameter of the growth inhibition zone around the well (measured in triplicate) was the parameter used to evaluate the antibacterial activity.

#### 2.3.9. Determination of Antioxidant Activity of CNSL

The methods of 2,2-diphenyl-1-picrylhydrazyl radical (DPPH), 2,2′-azino-bis3-ethylbenzothiazoline-6-sulfonic acid (ABTS) and total antioxidant capacity were performed to investigate the antioxidant activity of CNSL. The analyses were conducted in triplicate, and the percentage of the removed DPPH was used to determine inhibition activities [19]. The standard used was Trolox^®^ (A vitamin E analog). The percentage of inhibition (I%) was determined by Equation (1):

I% = [(Ac − As)/(Ac)] × 100(1)

where Ac is the control absorbance and As is the sample absorbance.

All tests were performed in triplicate. The IC50 of DPPH was determined by linear regression of the percentage of remaining DPPH compared to the concentration of the sample.

The antioxidant activity of CNSL by the ABTS test was determined considering the cationic chromophore radical from ABTS oxidation [20]. The measurements were performed in triplicate, and the percentage of the removed ABTS was used to calculate the inhibition activity. The standard used was Trolox^®^ (a vitamin E analog). The percentage of inhibition (I%) was determined as demonstrated previously. All the tests were performed in triplicate. The IC50 of ABTS was determined by linear regression of the percentage of remaining ABTS compared to the concentration of the sample.

The phosphomolybdenum method was used to investigate the total antioxidant capacity [21]. The assay was conducted considering the reduction in molybdenum+6 to molybdenum+5 by the sample and posterior generation of a greenish phosphate/molybdenum+5 complex. Tubes with both samples and reagents (0.6 M sulfuric acid, 28 mM sodium phosphate, and 4 mM ammonium molybdate) were incubated at 100 °C for 90 min. Subsequently, the absorbance of each solution was read at 695 nm against a blank solution. The reference used was ascorbic acid. The total antioxidant capacity (I%) was determined using the I% equation demonstrated previously. All tests were performed in triplicate. The IC50 of TAC was determined by comparing the regression activity of the coating to the concentration of the sample.

#### 2.3.10. Determination of the Antioxidant Activity of the Films

For the evaluation of the antioxidant activity of the biofilms, the method of capture of the DPPH radical (2,2-Diphenyl-1-picrylhydrazyl) was used from the adapted methodology of Blois [20] according to Souza et al. [8]. Rectangular samples of each film (20 mg) were transferred to tubes with 1 mL of DPPH methanolic solution (60 µM), and the mixture was then gently stirred for 30 min at room temperature (25 °C) and protected from light. Tubes containing only the DPPH solution were used as controls. Subsequently, 200 µL of the solution was placed into 96-well plates, and the absorbance was read at 515 nm (ELISA reader, Bio-Rad Laboratories Hercules, CA, USA) [22]. The free radical (%) elimination by the sample was determined by Equation (2):

DPPH elimination capacity = (Abs515control − Abs515sample)/Abs515control × 100(2)

where Abs515sample is related to the absorbance of the tubes with the alginate films with or without CNSL and Abs515control is related to the absorbance of the control tubes. All determinations were performed in triplicate, and the results are expressed as the mean ± standard deviation.

#### 2.3.11. Toxicity Test

The toxicity test of CNSL on *Artemia salina* was carried out according to Meyer et al. [23] using a solution with sea salt at 30 g/L. A 0.1 mol/L NaOH solution was used to adjust the pH between 8.0 and 9.0, which was also used for hatching the eggs of *Artemia salina* and in the preparation of the other solutions by dilution. The eggs were put in to hatch in saline solution for 48 h under aeration at room temperature (25 °C). Then, approximately 10 larvae of *Artemia salina* were placed into tubes with saline solution and samples with CNSL at 10, 100, 500, 1000, and 1500 ppm and crude extract of the hydrolate. The test was performed in triplicate, and the counting of dead and alive animals was performed after 24 h.

A simple linear equation was adjusted to the data on the percentage of dead *Artemia salina* larvae in relation to the increased CNSL concentration, which was used to estimate the concentration of extract responsible for killing 50% of the larvae. A graphic analysis method was used to obtain the LD50 (lethal dose of extract for 50% of the population). The test was accompanied by a negative control involving only saline solution.

### 2.4. Statistical Analysis

All tests were conducted in triplicate, and the results are expressed as the mean ± standard deviation. The means of the groups were compared by unidirectional analysis of variance (ANOVA) and then Tukey’s test with GraphPad Prism software. Statistical data were considered significant at *p* < 0.05.

## 3. Results

### 3.1. Thermogravimetric Analysis (TGA) and Differential Scanning Calorimetry (DSC)

**Figure 1:**
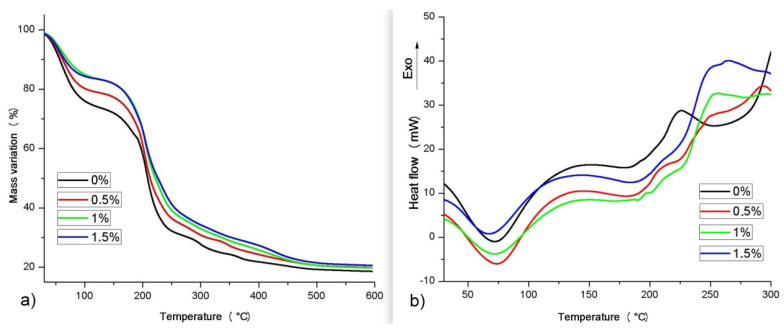
(**a**) Thermogravimetric (TG) and (**b**) differential scanning calorimetry (DSC) curves of the bioactive films obtained from the incorporation of cashew nut shell liquid into a sodium alginate matrix.

The thermogravimetric curves of the studied materials are shown in Figure 1a, where all the films presented two main events of thermal degradation.

The first event in the TGA curves is associated with moisture loss, with values of 26.6, 21.3, 16.6, and 16.7 for films with inclusions of 0%, 0.5%, 1%, and 1.5% CNSL, respectively. The second event concerns thermal degradation, and the initial temperature (T_onset_) was similar for all films (184, 183, 183, and 183 °C for 0%, 0.5%, 1%, and 1.5%, respectively). Films 0%, 0.5%, 1%, and 1.5% showed temperature ranges of thermal degradation of 129 to 270 °C, 124 to 269 °C, 126 to 288 °C, and 120 to 294 °C, respectively (Figure 1a).

Figure 1b shows the DSC curves of films. The 1st peak, at approximately 70 °C, is an endothermic event relative to moisture, as also observed in the TG curves. The 2nd peak corresponds to an exothermic event relative to the beginning of thermal degradation, with 227 °C for 0% CNSL and approximately 212 °C for the films, regardless of the CNSL inclusion. The films with CNSL showed a 3rd event, attributed to the main thermal degradation reaction, in which the inclusions of 0.5%, 1%, and 1.5% showed values of 249, 252, and 256 °C. The film with a 1.5% CNSL inclusion presented a 4th degradation event at 264 °C.

### 3.2. Morphology

**Figure 2:**
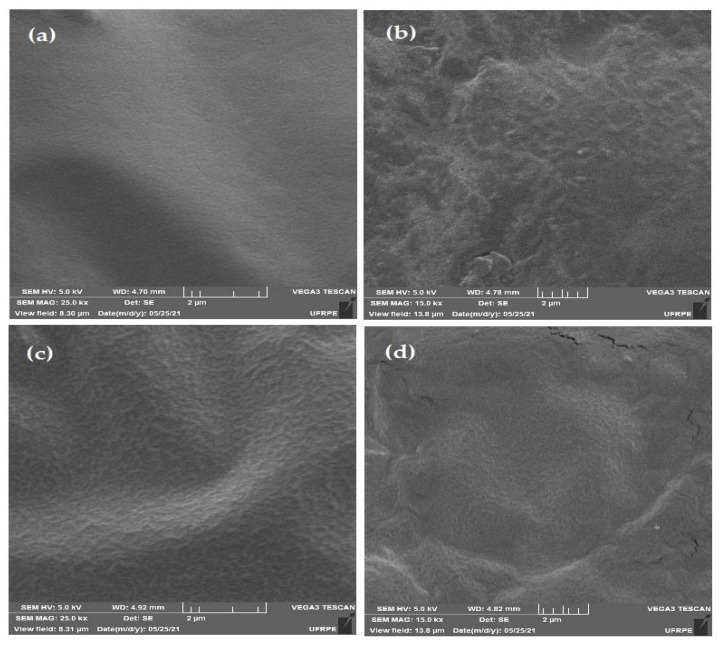
Surface microstructure of biopolymeric films enriched with different levels of cashew nut shell liquid (CNSL), including 0.5% (**b**), 1% (**c**), and 1.5% (**d**), in alginate matrix. Saline control (**a**).

The micrographs show that (Figure 2) the treatment without the addition of CNSL (a) presented a compact, smooth, regular microstructure, suggesting the formation of an ordered and homogeneous matrix, while the addition of CNSL (b, c, d) resulted in films that were generally similar to each other, with a slightly rougher surface than that in the treatment without oil inclusion (a). However, the films with the addition of 0.5% and 1% remained intact, with a sealed surface and no pores, while the incorporation of 1.5% CNSL (d) resulted in a film with the presence of cracks, indicating that this concentration made the film more brittle.

### 3.3. Color Parameters, Mechanical Proprieties, Water Vapor Permeability, and Thickness

With the analysis of the color of the presented films and the measure to which the concentrations of the CNSL increased in the films, we observe that the luminosity was reduced (*p* < 0.05), and the colors red, yellow, saturation, and opacity rate were increased, where the less luminous the films are, the more opaque they are (Table 1). All the films that have been enriched with CNSL tend to move the color toward red (positive values of parameter a*) and yellow, and where the greater proportion of the liquid is present, the tonality is more intense. However, for the saturation index or chroma and the yellow color, the treatments with 1.0% and 1.5% CNSL inclusion did not show a difference (*p* > 0.05).

The permeability increased as the level of CNSL increased (*p* < 0.05), especially for the concentrations of 1% and 1.5% CNSL, between which no significant difference was observed (*p* > 0.05). Regarding the mechanical properties of the films, the maximum tensile strength of the films decreased proportionally due to the addition of CNSL (*p* < 0.05), while the addition improved (*p* < 0.05) the elasticity of the films. Regarding film thickness, the inclusion of CNSL increased (*p* < 0.05) in proportion to the level of CNSL inclusion compared to that of the treatment without inclusion.

### 3.4. Antimicrobial Activity

The results shown in Table 2 regarding the antimicrobial activity of the CNSL included in the films reveal that the CNSL presents antimicrobial activity for all the strains tested (*S. typhimurium; E. coli; P. aeruginosa; L. monocytogenes; B. cereus;* and *S. aureus*).

When incorporated in the filmogenic solution (FS), the CNSL presented antimicrobial activity at inclusion in 1% of the films for the strains of *P. aeruginosa, L. monocytogenes, B. cereus,* and *S. aureus*, with no antimicrobial activity for the strains of *S. typhimurium* and E. *coli*, as described in Table 3. Furthermore, there was also a saturation of the effectiveness at the level of 1%, and as the level increased to 1.5%, there was no greater effectiveness in the elimination or control of the microorganisms.

Therefore, the antimicrobial activity of FS was indicated to be less efficient than the antimicrobial activity of technical CNSL without incorporation, especially for Gram-negative bacteria.

### 3.5. Antioxidant Activity

The CNSL showed potent antioxidant activity when compared to the control treatments (*p* < 0.05) according to three different evaluation methods (Table 4).

**Figure 3:**
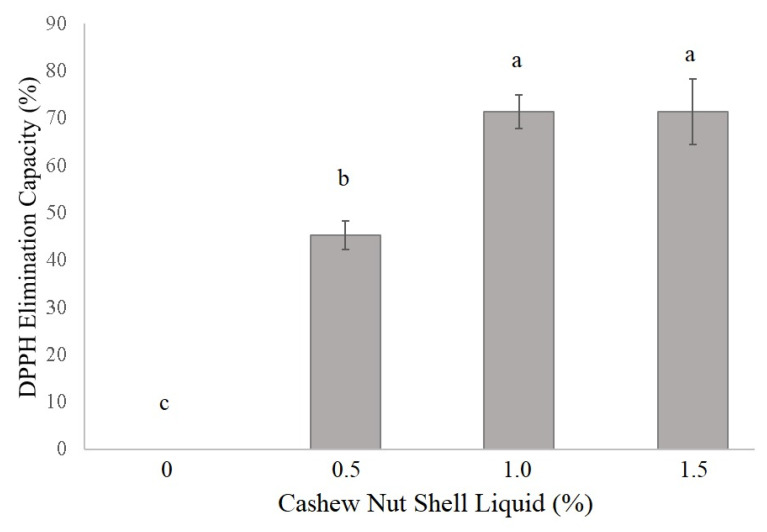
Antioxidant activity (elimination capacity of DPPH) of bioactive films obtained from the incorporation of cashew nut shell liquid (CNSL) into a matrix of sodium alginate. Mean ± standard deviation (*n* = 3), ^a–c^ means followed by different lowercase letters differ by the Tukey test (*p* < 0.05).

Regarding the antioxidant activity of CNSL-enriched alginate films, the inclusion of oil significantly increased (*p <* 0.05) the antioxidant activity of the films, with a DPPH elimination capacity of 0% without CNSL, 45% with the inclusion of 0.5% CNSL, and 71.4% in treatments with 1.0% and 1.5% of CNSL. There was no difference in DPPH elimination capacity between the 1.0% and 1.5% levels of CNSL inclusion (Figure 3).

### 3.6. Toxicity Test

**Figure 4:**
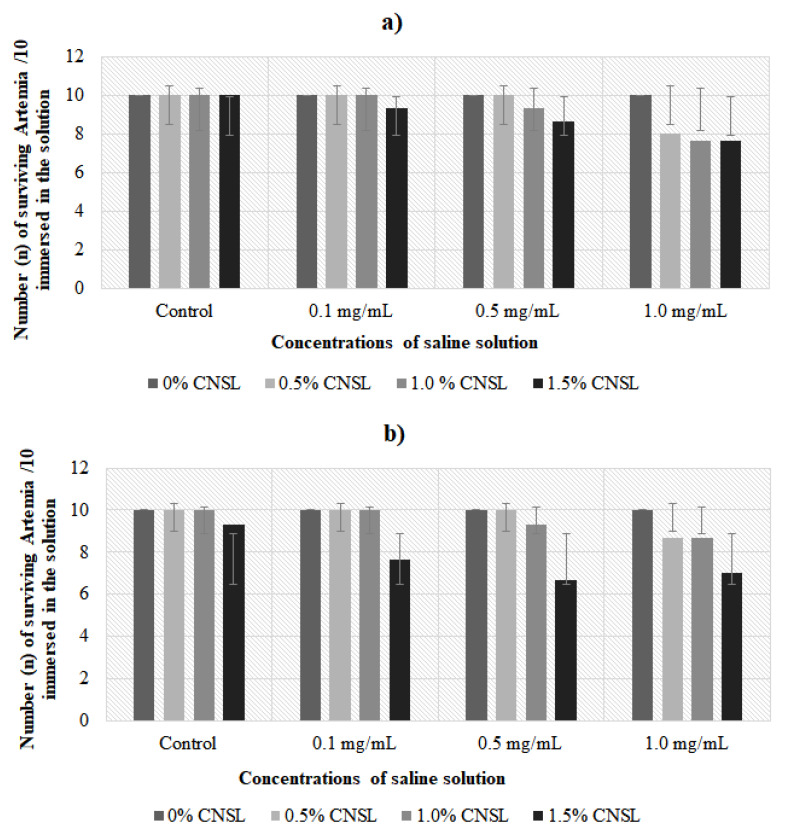
Determination of *Artemia* larva (*n* = 10) ecotoxicity in different salt concentrations from films enriched by different concentrations of cashew nut shell liquid (CNSL) after 24 (**a**) and 48 h (**b**). Values are described as the mean ± standard deviation (*n* = 3); SEM = standard error of the mean; difference by the Tukey test when *p* < 0.05.

Based on the number of live nauplii in ecotoxicity tests, it was possible to observe that the inclusion of CNSL up to 1.5% in biopolymer films to replace conventional packaging does not present health risks considering that, after 24 h, the number of live nauplii was greater than 70% at all concentrations studied and that after 48 h, more than 50% of nauplii remained alive, confirming the nontoxicity, as shown in Figure 4.

## 4. Discussion

In addition to the material for the production of biodegradable films, several parameters need to be considered, such as their thermal degradation [24], which varies according to the composition of each film [25]. The thermal resistance proportionally improved with the addition of CNSL, considering that the oil possesses a chemical structure capable of resisting high initial degradation temperatures, especially due to the presence of aromatic rings. Furthermore, due to its high flame resistance, CNSL has the capacity to improve the thermal resistance of other polymers and is an important characteristic for food films/packaging [26,27]. Considering that the moisture content decreases as the CNSL content increases, increased CNSL contributes to a higher hydrophobicity and greater microbiological stability. Prasad and Pillai [28] also observed that the high presence of cardanol as a prepolymer improves the thermal stability of other polymers. This phenomenon was also observed by Menon et al. [29], where the inclusion of CNSL improved the thermal stability of the natural polymer studied. The thermal degradation of the film without the addition of CNSL is within acceptable levels in the literature, with an initial thermal temperature similar to that found by Yang et al. [30]. Although the films with CNSL had similar initial degradation temperatures (T_onset_), TG curves indicated that the higher the level of CNSL, the better the thermal stability of the respective film, given the decrease in the rate of thermal degradation, while DSC curves confirmed that the thermal stability of the films was directly proportional to the CNSL level. In particular, the inclusion of 1.5% CNSL produced a 3rd thermal degradation reaction in addition to two events in the other films, corroborating the better thermal stability of the film with 1.5% CNSL.

The color parameter of the film is also an extremely important characteristic and can increase the likelihood of acceptance by consumers [31]. The linear increase in the red values (a*) and chroma (c*) obtained in the enriched films is due to the reddish-brown coloration attributed to CNSL during its polymerization process, a result of the presence of cardol [32].

The linear increase in the a* and b* indexes as the CNSL level was increased caused the increased opacity of the films and, consequently, the decreased luminosity, considering that as they become darker, their opacity increases [33]. This effect is positive from the point of view of food conservation since opaque films are a barrier to light and the oxidative deterioration induced by it when applied to food, thus helping to avoid the loss of nutrients and discoloration as well as preventing unpleasant flavors from being imparted to the food product [8]. However, transparency is an important aspect from a commercial point of view to provide a better presentation and visualization of the quality of the product [34]. The increase in the opacity in films with the incorporation of the oil occurs because the oil droplets are distributed throughout the polymeric matrix, providing the dispersion of light [35].

Another very important aspect to be analyzed is the morphological aspects used to assess the structure of films, such as homogeneity and the presence of pores, cracks, voids, and imperfections [36], which directly impact the potential for food conservation. The films based on sodium alginate were in perfect condition, as in the study by Galus and Lenart [37] and Aloui et al. [38], showing that this property is characteristic of films of this polymeric matrix.

The structural irregularities found in the films after the inclusion of the CNSL arose from the decrease in the homogeneity of the biopolymer matrix as a result of the addition of a lipid component with aromatic rings and hydroxyl groups that were naturally hydrophobic, which affected the hydrophilic property of the films. This result was expected, providing imperfections and/or free spaces between the polymers and consequently affecting the increase in the passage of water vapor [35], especially in the film whose inclusion of CNSL was 1.5%, where the presence of cracks confirmed that the percentage of CNSL was as high as necessary to maximize its beneficial properties and therefore does not present food safety concerns when used as food packaging. This scenario explains the results for WVP, given that this variable increased as the oil level increased, as well as results related to the mechanical tests, the primary aspect to be analyzed in biodegradable films because it reflects the durability of films and their ability to preserve the integrity of food [39]. The tensile strength decreased proportionally due to the addition of CNSL, mainly for the treatment with the addition of 1.5% CNSL, because the addition of CNSL, as a hydrophobic agent, to hydrophilic film, causes the film to show irregular structures, thus helping the formation of films with less chain mobility and, as a consequence, less resistance to rupture [40]. The addition of oil, in turn, can increase the flexibility and mobility/elongation of the films [41], as observed in this study.

These results corroborate other studies carried out by Han et al. [41] and Ahmed et al. [42], where the inclusion of an essential oil, such as a hydrophobic substance, hampered the intermolecular interaction process between the polymer chains, resulting in fewer tensile-resistant and plastic materials, leading to increased mobility and flexibility of the films.

For food application purposes, the increase in this WVP is not preferable, considering that the best films are those that present low water vapor permeability [33]. This capability is essential since it establishes the capacity of interaction between the films and water, protecting foods against dehydration or rehydration and thus may reduce the deterioration of food and consequently increase its shelf life [8].

The increase in film thickness by the inclusion of CNSL is justified, as the addition of oil has led to an increase in the solid content of the films, thus increasing their thickness in comparison to 0% CNSL [43]. Thus, we believe that the added hydrophobic substance becomes attached in the form of microparticles in the polymeric matrix, with a decrease in density and, consequently, an increase in thickness [44]. The inclusion of a hydrophobic substance also favored an increase in the thickness of films based on sodium alginate, as described by Mahcene et al. [45], highlighting, however, that film thickness is directly related to composition [46] as well as the method of preparation and drying conditions [37].

CNSL showed an antimicrobial effect for all strains tested in this study. However, the efficiency of the antimicrobial action of CNSL in the biofilms occurred only from its inclusion at 1%. Therefore, two justifying hypotheses were developed: the first hypothesis was that part of the oil’s properties may have evaporated in the film preparation stage, which requires drying at 45 °C for 24 h, and the second hypothesis was related to the type of CNSL used (technical), bearing in mind that even though cardanol presents antimicrobial activity, its efficiency is considerably inferior to the efficiency of anacardic acids, a major component of CNSL in nature [47]. Anacardic acid is mainly responsible for most of the antimicrobial properties [48]; thus, a greater quantity of the compound is necessary to preserve its activity in the films by means of cardanol and cardol. The sodium alginate-based film had no antimicrobial function against the tested pathogens, corroborating the studies by Han et al. [41] and Ngo et al. [49].

Furthermore, the technical CNSL evidenced promising antioxidant activity that held up after incorporation into bioactive films, as the inclusion level increased due to the presence of phenolic lipid compounds with long and unsaturated chains. The presence of aromatic rings in the CNSL affords film antioxidant properties; unsaturation within the long side chain of cardanol can be a valuable factor in the capture of free radicals [13] because these substances act as oxygen donors for peroxyl radicals, making the free radical reaction impossible [50]. However, the antioxidant activity of CNSL is not only due to the presence of cardanol but also cardol and anacardic acid [51]. This characteristic is very important because by acting as a free radical eliminator and hydrogen donor, as is the case with most of the active compounds of natural antioxidants, there is an inhibition of the generation and propagation of reactive species and free radicals [52]. Corroborating these results, several studies indicate that the inclusion of additives endowed with phenolic compounds, such as functional and/or essential oils, promotes a significant improvement in the antioxidant activity of biopolymeric films [38,53,54].

The greater efficiency of the application of antioxidant packaging concerns foods with high fat content [38], as it delays lipid oxidation, which is the main cause of food deterioration, especially of the most perishable foods, and may directly affect their organoleptic qualities, such as color, flavor, and odor [39]. Oxidative reactions are responsible for reducing the nutritional value of foods through the degradation of essential fatty acids, proteins, and liposoluble vitamins, producing strange flavors and odors and promoting changes in food color due to pigment degradation [55].

Finally, the films developed in this study were nontoxic for the levels tested, which was expected because, in addition to the percentage incorporation being low, the CNSL used was technical grade, obtained by the processing and heating of the nuts at 180–200 °C, where anacardic acid suffers a decarboxylation reaction in which it is converted to cardanol [56] and becomes nontoxic. In addition, anacardic acid is responsible for the caustic, irritant, and toxic properties of the CNSL [57]. Cardanol is nontoxic [18]. Nevertheless, cardol, a compound present at a small concentration in CNSL (10%), shows a structure similar to that of anacardic acids, as it presents a second hydroxyl in the aromatic ring and is considered to have toxic properties in some studies [58], justifying the performance of the test in this study.

## 5. Conclusions

The inclusion of CNSL in the production of biopolymeric films based on sodium alginate proved to be a promising option for use in the food industry, especially due to its high antioxidant activity, which is maintained after inclusion in biopolymeric films, and its antimicrobial activity, evidenced by the inclusion of 1% CNSL. Additionally, the inclusion of CNSL is a sustainable option and can be used in the agrifood industry to replace nondegradable food packaging while improving the quality and increasing the shelf life of perishable food. Thus, we recommend the use of film with a CNSL inclusion of 1%, considering that this level of inclusion presented the best results in most of the analyses, with greater stability compared to that resulting from the 1.5% inclusion due to the increase in saturation level.

## Figures and Tables

**Table 1:** Color parameters, mechanical properties, and water vapor permeability (WVP) of the bioactive films obtained from the incorporation of cashew nut shell liquid (CNSL) into a matrix of sodium alginate.

Variables	Cashew Nut Shell Liquid (%)			
	0	0.5	1.0	1.5
Coloration index				
Luminosity (L*)	91.59 ± 1.02 ^a^	80.80 ± 1.10 ^b^	65.83 ± 3.95 ^d^	61.57 ± 2.02 ^c^
Redness (a*)	−1.10 ± 0.14 ^d^	1.61 ± 0.24 ^c^	6.73 ± 0.67 ^b^	9.34 ± 0.45 ^a^
Yelowness (b*)	7.01 ± 0.96 ^c^	16.9 ± 0.69 ^b^	24.00 ± 0.61 ^a^	24.03 ± 0.23 ^a^
Chroma (C*)	7.09 ± 0.97 ^c^	17.07 ± 0.70 ^b^	24.76 ± 0.63 ^a^	25.75 ± 0.31 ^a^
Opaciy	13.89 ± 0.34 ^d^	15.84 ± 0.91 ^c^	18.12 ± 0.75 ^b^	20.10 ± 0.98 ^a^
Mechanical properties				
Thickness (mm)	0.090 ± 0.02 ^b^	0.133± 0.04 ^a^	0.238± 0.04 ^a^	0.263±0.02 ^a^
Tensile strength (MPa)	54.71 ± 0.20 ^a^	41.54 ± 0.08 ^c^	44.03 ± 0.07 ^b^	36.58± 0.07 ^d^
Modulus of elasticity (MPa)	95.75 ± 0.36 ^a^	72.70 ± 0.14 ^c^	77.04 ± 0.12 ^b^	64.01± 0.13 ^d^
Elongation at break (%)	42.26 ± 3.94 ^b^	38.23 ± 2.94 ^b^	49.78 ± 4.67 ^a^	51.07 ± 1.88 ^a^
WVP [10^−10^ g.(m.s.Pa)^−1^]	7.16 ± 0.30 ^c^	9.11 ± 0.19 ^b^	29.12 ± 7.11 ^a^	31.02 ± 3.24 ^a^

Mean ± standard deviation, ^a–d^ means followed by different lowercase letters differ by the Tukey test (*p* < 0.05).

**Table 2:** Antimicrobial activity of bioactive films obtained from the incorporation of cashew nut shell liquid (CNSL) into a matrix of sodium alginate expressed as minimum inhibitory concentration (MIC) and minimum bactericidal concentration (MBC).

Strains	Cashew Nut Shell Liquid (µg/mL)		Azithromycin (µg/mL)	
	MIC	MBC	MIC	MBC
*S. typhimurium*	128	256	2	4
*E. coli*	512	nd	32	16
*P. aeruginosa*	128	256	8	16
*L. monocytogenes*	128	256	8	16
*B. cereus*	128	256	4	8
*S. aureus*	32	64	8	16

^nd^ Not determined.

**Table 3:** Antimicrobial activity of bioactive films obtained from the incorporation of cashew nut shell liquid (CNSL) into a matrix of sodium alginate in different concentrations using the well diffusion agar method expressed as inhibition area diameter (mm).

Microorganisms	Filmogenic Solutions (CNSL)				Controls	
	0%	0.5%	1.0%	1.5%	Azithromycin	Water
*S. typhimurium*	NI	NI	NI	NI	21.6 ± 0.57	NI
*E. coli*	NI	NI	NI	NI	16.3 ± 0.57	NI
*P. aeruginosa*	NI	NI	7.3 ± 1.15^c^	10.6 ± 0.57^b^	18.3 ± 0.57^a^	NI
*L. monocytogenes*	NI	NI	7.5 ± 0.50^c^	11.5 ± 0.50^b^	18.5 ± 0.50^a^	NI
*B. cereus*	NI	NI	6.5 ± 0.50^b^	7.6 ± 0.5^b^	18.6 ± 0.57^a^	NI
*S. aureus*	NI	NI	9.6 ± 0.57^c^	11.3 ± 0.57^b^	20.3 ± 0.57^a^	NI

NI = Not identified; Mean ± standard deviation, ^a–d^ means followed by different lowercase letters differ by the Tukey test (*p* < 0.05).

**Table 4:** Antioxidant activity expressed as the IC_50_ (µg/mL) of cashew nut shell liquid (CNSL).

Sample	DPPH	ABTS	TAC
CNSL	132.89 ± 0.27 ^a^	102.38 ± 0.31 ^b^	1482.81 ± 0.44 ^b^
Trolox	28.13 ± 0.11 ^b^	153.67 ± 0.02 ^a^	N.T.
Ascorbic acid	N.T.	N.T.	50000 ± 0.01 ^a^

Mean ± standard deviation (*n* = 3), ^a,b^ means followed by different lowercase letters differ by the Tukey test (*p* < 0.05). DPPH: 2,2-Diphenyl-1-picryl-hydrazyl radical; ABTS: 2’,2-Azino-bis(3-ethylbenzothiazoline-6-sulfonate) radical; TAC: total antioxidant capacity; N.T.: not tested.

## Data Availability

The data presented in this study are available in article.
